# Insulin-Like Growth Factor 1 Receptor and Response to Anti-IGF1R Antibody Therapy in Osteosarcoma

**DOI:** 10.1371/journal.pone.0106249

**Published:** 2014-08-29

**Authors:** Yu Cao, Michael Roth, Sajida Piperdi, Kristofer Montoya, Rebecca Sowers, Pulivarthi Rao, David Geller, Peter Houghton, E. Anders Kolb, Jonathan Gill, Richard Gorlick

**Affiliations:** 1 Division of Pediatric Hematology/Oncology, Children’s Hospital at Montefiore, Albert Einstein College of Medicine, Bronx, New York, United States of America; 2 Division of Pediatric Hematology/Oncology, Texas Children’s Cancer Center, Baylor College of Medicine, Houston, Texas, United States of America; 3 Department of Orthopedic Surgery, Montefiore Medical Center and the Children’s Hospital at Montefiore, Albert Einstein College of Medicine, Bronx, New York, United States of America; 4 Center for Childhood Cancer and Blood Diseases, Nationwide Children’s Hospital, Columbus, Ohio, United States of America; 5 Division of Pediatric Hematology/Oncology, A.I. duPont Hospital for Children, Wilmington, Delaware, United States of America; 6 Department of Molecular Pharmacology, Albert Einstein College of Medicine, Bronx, New York, United States of America; Johns Hopkins University, United States of America

## Abstract

**Background:**

Survival outcomes for patients with osteosarcoma (OS) have remained stagnant over the past three decades. Insulin-like growth factor 1 receptor (IGF1R) is over-expressed in a number of malignancies, and anti-IGF1R antibodies have and are currently being studied in clinical trials. Understanding the molecular aberrations which result in increased tumor response to anti-IGF1R therapy could allow for the selection of patients most likely to benefit from IGF1R targeted therapy.

**Methods:**

*IGF1R* mRNA expression was assessed by RT PCR in OS patient primary tumors, cell lines, and xenograft tumors. *IGF1R* copy number was assessed by 3 approaches: PCR, FISH, and dot blot analysis. Exons 1–20 of *IGF1R* were sequenced in xenograft tumors and 87 primary OS tumors, and surface expression of *IGF1R* was assessed by flow cytometry. Levels of mRNA and protein expression, copy number, and mutation status were compared with tumor response to anti-*IGF1R* antibody therapy in 4 OS xenograft models.

**Results:**

*IGF1R* mRNA is expressed in OS. Primary patient samples and xenograft samples had higher mRNA expression and copy number compared with corresponding cell lines. *IGF1R* mRNA expression, cell surface expression, copy number, and mutation status were not associated with tumor responsiveness to anti-IGF1R antibody therapy.

**Conclusions:**

*IGF1R* is expressed in OS, however, no clear molecular markers predict response to IGF1R antibody-mediated therapy. Additional pre-clinical studies assessing potential predictive biomarkers and investigating targetable molecular pathways critical to the proliferation of OS cells are needed.

## Introduction

Osteosarcoma (OS) is the most common primary bone malignancy in children and young adults [1]. Current treatment strategies have achieved a long-term survival rate of approximately 70% in patients with localized disease at presentation [1], [2]. Unfortunately patients with metastatic or relapsed disease have extremely poor prognoses. There has been minimal improvement in outcomes over the past three decades [1], [2]. Novel therapies are needed to improve survival for these patients.

Treatment strategies that target biological pathways driving the proliferation and survival of the malignant cells have recently proven successful in hematologic and solid malignancies. The efficacy of trastuzumab for patients with breast cancer, and imatinib for patients with chronic myelogenous leukemia and gastrointestinal stromal tumor has encouraged researchers to identify targetable pathways essential for cancer cell pathophysiology [3]–[5]. The insulin-like growth factor (IGF) pathway is important for regulating cellular growth, proliferation, and stress response in both normal tissue and cancer cells [6]. High expression of insulin-like growth factor 1 receptor (IGF1R) and its two ligands, insulin-like growth factor 1 (IGF1) and insulin-like growth factor 2 (IGF2) have been demonstrated in OS, as well as many other cancers including rhabdomyosarcoma, breast cancer, prostate cancer, and colon cancer [7]–[14]. IGF1R is a cell-surface receptor tyrosine kinase which forms a homo-dimer upon binding with its ligand, IGF1 or IGF2. IGF1R then auto-phosphorylates which leads to the activation of downstream signaling cascades including the PI3K–AKT–TOR and the RAF–MAPK pathways. These signaling cascades have been shown to stimulate cell survival mechanisms, inhibit apoptosis, result in enhanced protein synthesis, and promote cell proliferation [6], [15]. *In vitro* studies demonstrate that IGF1 rescues cancer cells from chemotherapy-induced apoptosis, and high expression is associated with a metastatic phenotype [6], [16], [17].

Inhibitors of *IGF1R* and its downstream pathways have shown promise in preclinical models of OS [1], [1]–[22]. Clinical trials of *IGF1R*-inhibiting antibody therapies in patients with sarcomas, however, have returned mixed results: patients show variability in responsiveness to these therapies [23], [24]. The biologic basis for differences in response to anti-IGF1R therapy is unclear. We hypothesized that genetic alterations in *IGF1R*, such as amplifications and mutations, may impact response to treatment.

## Methods

### Patient Samples, Xenograft Samples, and Cell Culture

OS primary tumors were collected at Memorial Sloan-Kettering Cancer Center (New York, NY) and Montefiore Medical Center (Bronx, NY) after obtaining written informed consent according to a biology study approved by the Memorial Sloan-Kettering Cancer Center IRB and the Montefiore Medical Center IRB. All samples were confirmed to have a pathologic diagnosis of OS. CB-17 SCID mouse (Taconic, Germantown, NY) xenografts were established from OS patient samples by the Pediatric Preclinical Testing Program (PPTP) as described previously.^19^ All xenograft experiments were performed in accordance with protocols approved by the Institutional Animal Care and Use Committee of the Albert Einstein College of Medicine. Corresponding cell lines were developed from selected primary tissue samples by standard collagenase disaggregation. All isolated cells were maintained as a monolayer in MEM-α media (Lonza, Allendale, NJ) supplemented with 20% fetal bovine serum (Hyclone lab, Logan, Utah), 1 mM sodium pyruvate, 1% non-essential amino acid, and 1% pen-strep in a 5% CO_2_ humidified atmosphere at 37°C. The OS cell line, 143B, and the breast cancer cell line, MCF7, were purchased from ATCC (Manassas, VA), and the mesenchymal stem cells (MSCs) from Lonza (Allendale, NJ). All cells were cultured as per the manufacturers’ instructions. The cell line corresponding to xenograft model M2 was not used secondary to overgrowth of murine fibroblasts within the cell line when grown *in vitro*.

### IGF1R Gene Expression Studies

Total RNA was extracted from the same set of samples using PureLink RNA Mini Kit (Life Technologies, Carlsbad, CA), and subsequently converted to cDNA using SuperScript III First-Strand Synthesis System (Life Technologies, Carlsbad, CA) according to the manufacturers’ instructions. Gene expression quantitation was carried out using a 7500 Fast Real-Time PCR system and Taqman Gene Expression assay mix (Life Technologies, Carlsbad, CA; Assay ID: Hs00181385_m1). The house keeping gene *GAPDH* was used as an endogenous control normalized to each sample for its mRNA content and multiple wells of scrambled control were included as negative controls. Reactions for each sample were done in triplicate for both *IGF1R* and *GAPDH*. Sample mRNA levels were relatively quantified using the ΔΔCT method as described in 7500 Sequence Detection System (Life Technologies, Carlsbad, CA). MSCs were used as the calibrator. Results are reported as the mean plus or minus standard deviation. Student t test was utilized to determine statistical significance between mRNA expression levels in the primary OS samples, primary xenograft samples, and the OS cell lines. P value less than 0.05 was considered statistically significant. Pearson’s r was utilized to determine the correlation between mRNA expression levels in primary OS samples compared with OS cell lines.

### IGF1R Gene Sequencing

Genomic DNA from 64 OS primary samples and 4 xenograft OS tissues were extracted using the QIAamp DNA Mini Kit (Qiagen, Valencia, CA). PCR primers were designed using Primer3 software, and purchased from IDT DNA (Coralville, IA). Primer sequences are provided in Table S1. The PCR primers also served as sequencing primers. PCR products were obtained for each of the 21 exons comprising *IGF1R* and verified by agarose gel. PCR products were then purified using the QIAquick PCR Purification Kit (Qiagen, Valencia, CA), and sequenced in both the forward and reverse directions using BigDye Terminator v3.1 Cycle Sequencing Kit (Life Technologies, Carlsbad, CA) and data was generated with an ABI 3100 sequencer (Applied Biosystems, Foster City, CA).

### IGF1R Copy Number Assays

Genomic DNA from all samples and cell lines were extracted as described above and quantitated using Taqman RNaseP Detection Reagents (Life Technologies, Carlsbad, CA). To determine the *IGF1R* copy number, quantitative PCR was performed using three different Taqman copy number (CN) assays (Life Technologies, Carlsbad, CA; Assay IDs: Hs00401826_cn, Hs01239357_cn, Hs02543373_cn) targeting different locations on the chromosome 15 where the *IGF1R* gene spans. According to NCBI build 37 database, the three CN assay locations were at chr15∶99251313 (overlaps Exon 2 - Intron 2), chr15∶99460087 (overlaps Exon 10 - Intron 10) and chr15∶99491821 (overlaps Intron 19 - Exon 20), respectively. *RNaseP* was used as the reference internal control. Data analysis was done using CopyCaller software (Life Technologies, Carlsbad, CA), with the MSCs as the normal control.

### Fluorescence *in situ* Hybridization (FISH) Studies

Fluorescence in situ hybridization (FISH) was performed to detect *IGF1R* gene amplification on 4 OS xenograft cell lines and MSC. Samples were incubated overnight with colcemid to arrest division at metaphase/interphase. Cells were then placed in hypotonic solution (0.4% KCl) for 10 minutes and fixed to slides using 3∶1 (methanol:acetic acid) at 22C and 50–60% humidity. Slides were hybridized overnight with *IGF1R* probe (15q26) and the control probe (15q11) (Veridex, North Raritan, NJ), stained with ProLong Gold antifade with DAPI (Life Technologies, Carlsbad, CA), and viewed under Upright Olympus BX61 fluorescent microscope at 60× power. Images were acquired using Cooke Sensicam cooled CCD camera and IP lab 4.0.8 software. Selected OS cell line samples and MSCs were also sent to Dr. Pulivarthi Rao (Cancer Cytogenetics Core Laboratory, Texas Children’s Cancer Center and Hematology Center, Baylor College of Medicine, Houston, TX) for validation of the FISH results.

### Dot-blot Experiments

Dot-blot experiments were performed to confirm *IGF1R* copy number results from the Taqman assay and FISH approaches. Full-length cDNA for *IGF1R* and Galactose-1-phosphate uridyliltransferase (*GALT*) were purchased from Origene (Rockville, MD). The DNA was labeled with digoxigenin (DIG) using the DIG High Prime DNA Labeling and Detection kit (Roche Applied Science, Indianapolis, IN). *GALT* served as a quantitative loading control. Optimum labeling efficiency of *IGF1R* and *GALT* probes were determined according to the manufacturer’s protocol. Serially diluted genomic DNA was fixed to a nylon membrane by cross linking with UV-light. The membrane was incubated with maleic acid buffer and blocking solution, and then hybridized with the DIG-labeled *IGF1R* DNA probe at 4°C overnight. CSPD substrate (Roche Applied Science, Indianapolis, IN) was used for chemiluminescent detection and developed on x-ray film. The *IGF1R* probe was subsequently stripped with the standard stripping buffer and re-hybridized with DIG-labeled *GALT* probe. Dot-blot size and density were measured using NIH ImageJ densitometry software.

### Flow Cytometry Studies

Four OS xenograft models from the Pediatric Preclinical Testing Program were stained with CFS-conjugated anti-human IGF1R monoclonal antibody (R&D systems, Minneapolis, MN) according to the manufacturer’s protocol. Flow cytometry analysis was performed using a BD LSRII digital bench top flow cytometer (Becton Dickinson, Mountain View, CA). Analysis was performed with FloJo software (Tree Star Inc., Ashland, OR). MCF7 cells were used as the positive control and the isotype control was used as the negative control in the flow cytometry analysis. Experiments were performed in triplicate. Results are reported as the mean fluorescent intensity (MFI) plus or minus standard deviation. Student t test was utilized to determine statistical significance of the difference in the mean fluorescent intensity between the OS xenograft models. P value less than 0.05 was considered statistically significant.

### Xenograft Models

**Figure 1:**
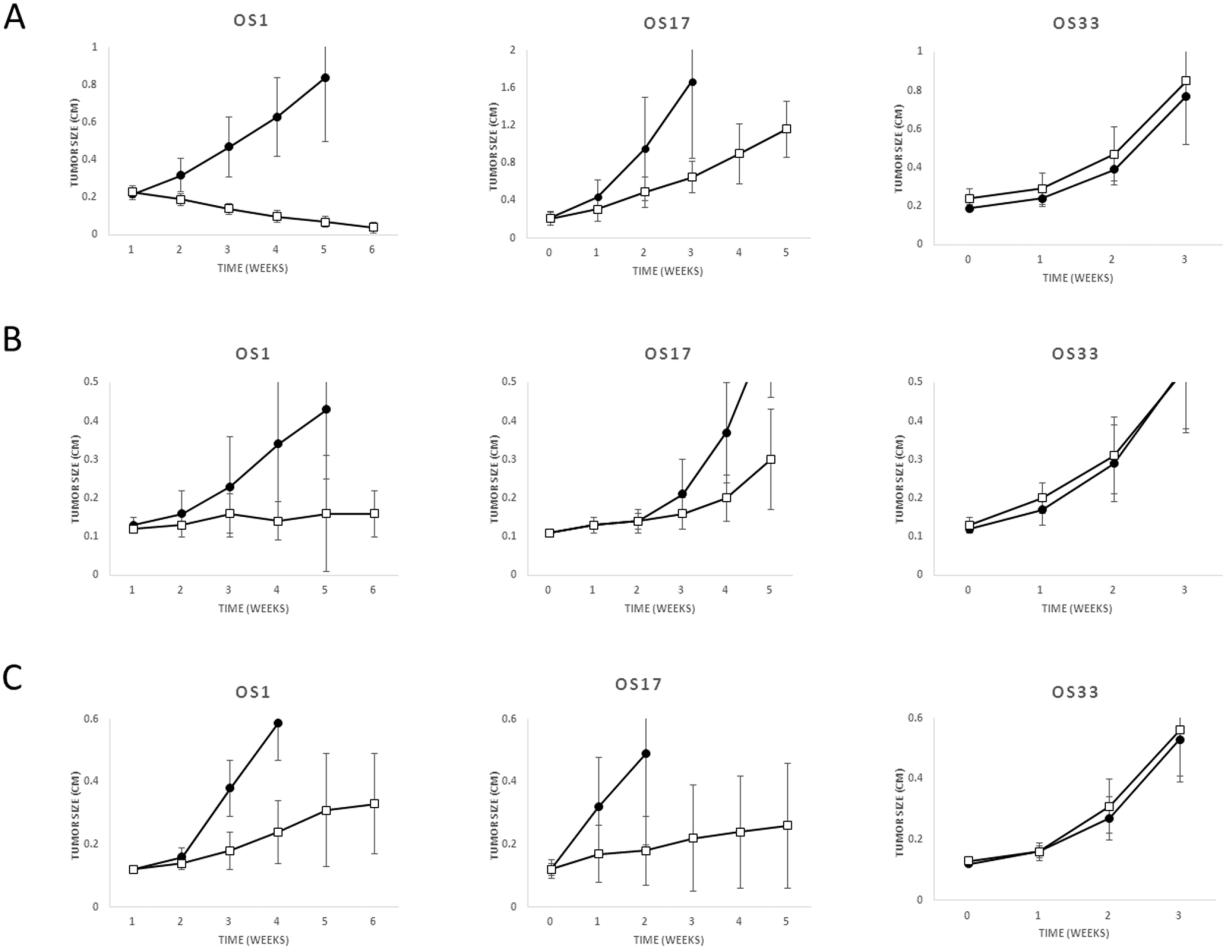
OS Xenograft Model Response to anti-IGF1R Antibody SCH 717454 Treatment. Three OS xenograft models were treated with an anti-IGF1R antibody SCH 717454 (A) (open square) or vehicle (closed circle), small molecule IGF1R inhibitor BMS 754807 (B) (open square) or vehicle (closed circle) or IMC A12 (C) (open square) or vehicle (closed circle). Mice were treated with 0.5 mg SCH 717454 administered twice weekly via intraperitoneal injection for 4 weeks, BMS 754807, 25 mg/kg administered orally BID for 6 days, repeated for 6 weeks, or IMC A12, 1 mg/mouse administered intraperitoneally twice weekly for 6 weeks. These studies were previously reported; however, the figures for M17 and M31 were not published [14], [19], [20]. Ten mice were included in each arm of the experiments. Tumor size was monitored weekly for a maximum of 6 weeks of treatment or until a relative tumor volume increased to 4× baseline.

Four OS xenograft models from the Pediatric Preclinical Testing Program were treated with anti-IGF1R antibody or a small molecule IGF1R inhibitor in prior studies [19], [21], [22]. Tumor response to anti-IGF1R antibody SCH 717454 treatment (0.5 mg administered twice weekly via intraperitoneal injection for 4 weeks), small molecule IGF1R inhibitor BMS 754807 (25 mg/kg administered orally BID for 6 days, repeated for 6 weeks), and anti-IGF1R antibody IMC A12 (1 mg/mouse administered intraperitoneally twice weekly for 6 weeks) were stratified into high responders (M1), intermediate responders (M2 and M17), and poor responders (M31) based on published prior studies [14], [19], [20]. Ten mice were included in each arm of the experiments. Figures demonstrating OS xenografts M17 and M31 tumor response to SCH 717454, BMS 754807, and IMC A12 have not been previously published and are shown in Figure 1; however, the data was obtained and summarized as part of the prior studies [14], [19], [20].

## Results

### OS Xenograft Tumor Response

Osteosarcoma xenograft models OS1, OS17, and OS31 were treated with monotherapy anti-IGF1R therapies SCH 717454, BMS 754807, and IMC A12 in previous PPTP studies [14], [19], [20]. Summarizing the previously reported data, M31 did not demonstrate tumor inhibition to any of the anti-IGF1R antibodies. (Figure 1) M1 demonstrated the greatest response to therapies SCH 717454 and BMS 754807, with near complete regression of tumors in response to antibody SCH 717454 (mean tumor volume in anti-IGF1R antibody arm vs placebo arm at 5 weeks, 1.09 cm^3^ vs. 0.04 cm^3^, p<0.001). M17 demonstrated an intermediate response to all 3 antibodies (Figure 1).

### IGF1R Gene Expression

**Figure 2:**
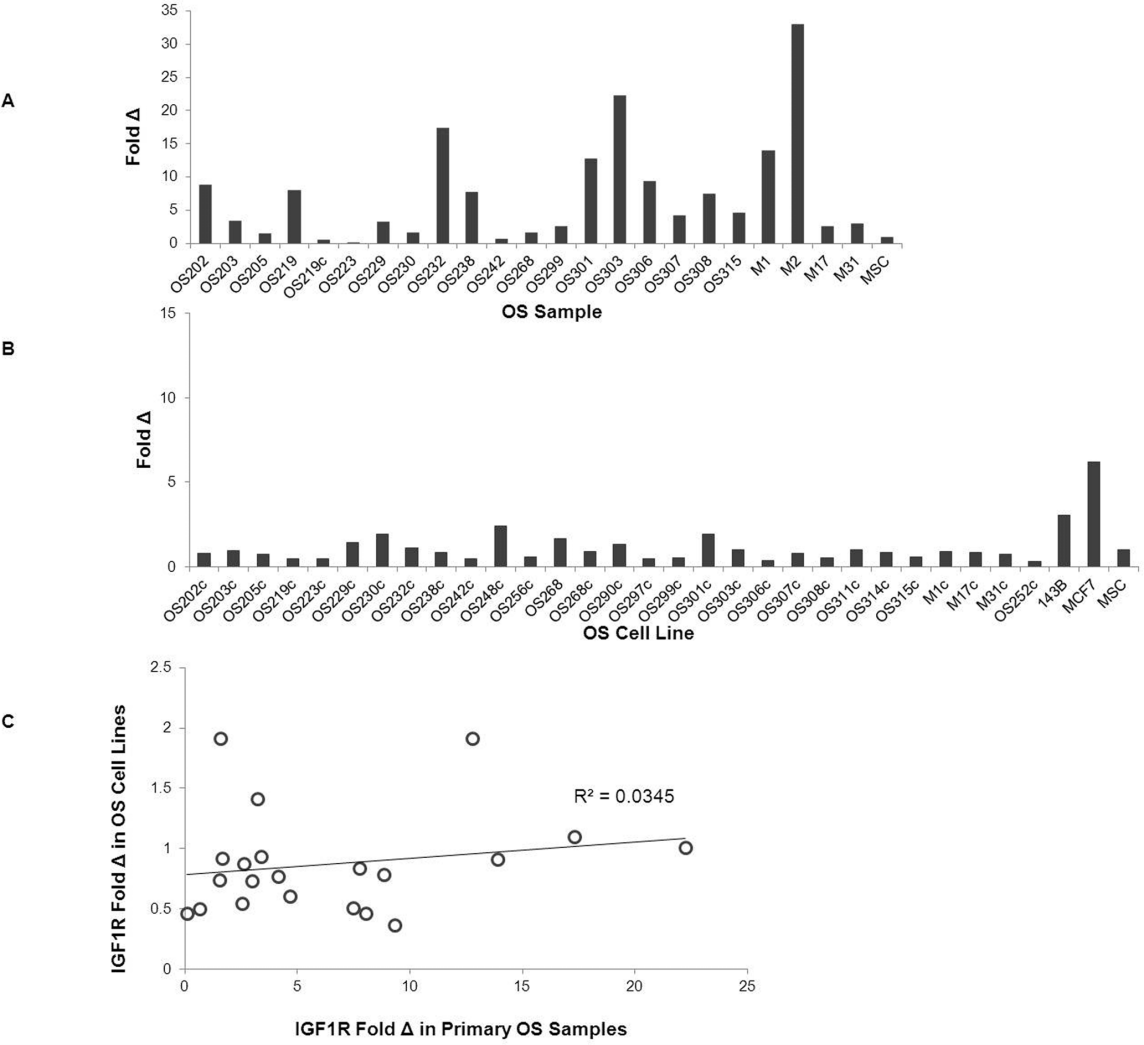
Relative IGF1-R mRNA Gene Expression in OS Primary Samples and Corresponding Cell Lines. Quantitative Real Time PCR detecting IGF1R mRNA expression was performed in OS primary samples (A) and OS cell lines (B). Grey bars represent relative quantification of IGF1-R expression using the ΔΔCT method using GAPDH as an endogenous control and MSC as a calibrator. Scatter plot comparing IGF1-R expression fold change in primary OS samples and their corresponding cell lines (C).

*IGF1R* mRNA expression was variable among the primary OS samples, primary xenograft samples, and the OS cell lines. IGF1R expression was higher in the primary patient specimens as compared to the OS cell lines (mean relative quantification 6.5+/−6.0 vs. 0.9+/−0.5, p<0.001). Levels of expression between the OS specimens did not correlate with the corresponding tumor derived cell lines (pearson’s r = 0.18). (Figure 2) Among the primary xenograft samples, *IGF1R* expression was higher in M1 and M2 compared with M17 and M31 (mean relative quantification 23.4+/−13.5 vs. 2.8+/−0.2, p = 0.28). Tumor response to treatment with anti-IGF1R antibody in the xenograft models was not clearly associated with total *IGF1R* mRNA expression.

### IGF1R Gene Alterations

Exons 1–20 of the IGF-1R gene were sequenced in OS samples: 4 xenograft tumors (M1 (high responder), M2 and M17 (intermediate responders), M31 (poor responder)) and 24 OS primary tumors. M1 had a sequence alteration in exon 16 (3179 G>A) that was not present in any of the low or intermediate responder xenograft samples. This sequence alteration results in a non-coding change. Exon 16 was subsequently sequenced on an additional 63 primary tumor samples. Of the total 87 OS primary tumor samples, 34 samples (39%) were homozygous wild type (GG), 33 samples (38%) were heterozygous (GA), and 20 samples (23%) were homozygous polymorphic (AA). Additional sequence alterations were present in the OS primary tumor samples, but these did not correlate with differences in response to treatment with anti-IGF1R antibody in the xenograft models. Real-time PCR was performed on 64 of the 87 OS tissue samples. There was no association between genotype and *IGF1R* expression. Mean fold change of IGF1R in tissue samples relative to HOS cells for genotypes GG, GA, AA were 4.11, 2.36, and 1.57, respectively (p = 0.6). Thus, the non-coding alteration did not impact *IGF1R* expression.

### IGF1R copy number

**Figure 3:**
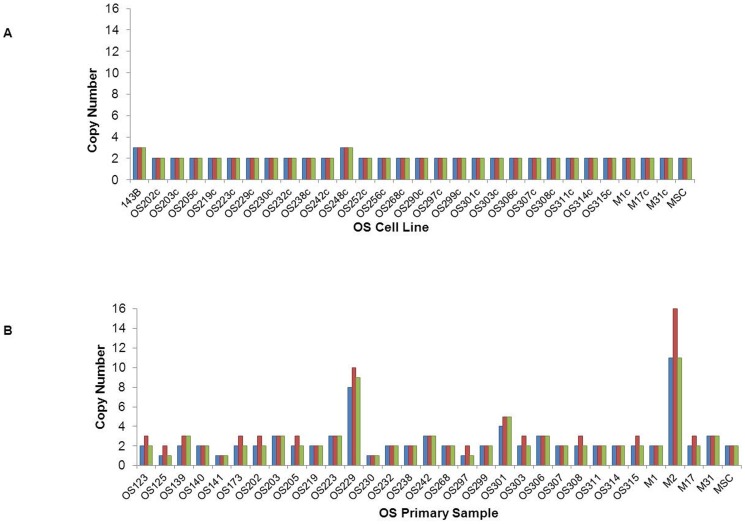
IGF1R Copy Number Assessed by PCR. IGF1R copy number was assessed in OS cell lines (A) and OS primary samples (B). MSCs were set to 2 copies of *IGF1R* and used as the calibrator. Three different copy number assays (blue, red, and green bars) were performed as described in the methods. Copy number is rounded to the nearest whole number. Blue bars represent results for the assay located at chr15∶99491821 (overlaps Intron 19 - Exon 20). Red bars represent results for the assay located at chr15∶99460087 (overlaps Exon 10 - Intron 10). Green bars represent results for the assay located at chr15∶99251313 (overlaps Exon 2 - Intron 2).(OS) signifies nucleic acids were derived directly from a human sample, (M) signifies cells were derived from a human tumor grown as a mouse xenograft, and (c) signifies the cells were passaged in cell culture.”

Three approaches were used to assess *IGF1R* copy number: quantitative PCR, FISH, and dot blot analysis. In the primary OS samples, PCR analysis demonstrated that while two patient-derived samples (229, 301) and M2 consistently showed amplification of (>4 copies) *IGF1R*, amplification of the *IGF1R gene was not detected in* the majority of the samples assayed. (Figure 3) Utilizing FISH, M1, M2, M17, and M31 were all found to have amplified IGF1R with 3–6, >5, 5–7, and 3–6 copies, respectively. (Table 1) Utilizing the dot-blot technique M2, M17, and M31 all showed amplification in *IGF1R*. (Table 1) Via dot-blot analysis, most cell lines did not show amplification of *IGF1R*, consistent with the data obtained by PCR. No clear association was seen in xenograft response to anti-*IGF1R* antibody and *IGF1R* copy number.

**Table 1:** Summary of IGF1R mRNA Expression and Copy Number in OS Xenograft Models.

Cell Line	Gene Expression(fold Δ compared to GAPDH)	Copy # PCR	Copy # FISH	Copy # Dot-Blot	Xenograft Response tomAb against IGF-1R
M1	13.9	2	3–6	N/A	+++
M1c	0.91	2		2	
M2	32.94	11–16	>5	4	+
M17	2.63	2	5–7	4	+
M17c	0.87	2		2	
M31	2.98	3	3–5	6	–
M31c	0.74	2		2	
MSC	1	2	2	2	not tested

### Cell Surface Expression of IGF1R

**Figure 4:**
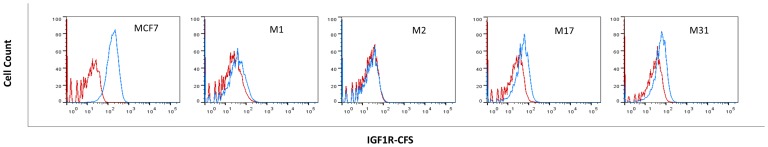
IGF1R Cell Surface Expression in OS Xenograft Models. FACS analysis of IGF1R surface expression in 4 OS xenograft models (M1: high responder, M2: intermediate responder, M17: intermediate responder, M31: high responder) and a positive control (MCF7) (A). The isotype control is represented by a red line and cells stained with IGF1R antibody are represented by a blue line.

Cell surface expression of IGF1R was assessed by flow cytometric analysis in the 4 xenograft models. MCF-7 cells were used as the positive control and demonstrated an MFI = 160.3+/−5.1, significantly higher than the MFI in all of the OS xenograft models. M31 (low responder) demonstrated the highest surface expression of IGF1R; while M1 (high responder) demonstrated significantly lower expression of surface IGF1R (M31 MFI = 42.9+/−6.5 vs M1 MFI = 25.6+/−0.4, p = 0.04). (Figure 4) M17 (intermediate responder) also demonstrated higher cell surface expression of IGF1R than M1 (high responder) (M17 MFI = 37.9+/−2.1 vs M1 MFI = 25.6+/−0.4, p<0.01). No clear association was seen in response to anti-*IGF1R* antibody and level of IGF1R cell surface antigen.

## Discussion

The prognosis for patients with metastatic and recurrent OS remains poor. Targeted therapies, attacking pathways that are essential for tumor growth and metastasis, provide possible avenues for improving the outcomes for these patients. In this study, alterations in the *IGF1R* gene were assessed by mutational analysis, copy number changes, mRNA expression, and cell surface expression in OS cell lines, human primary tumor samples, and xenograft tumor samples. The results were compared with the response to *IGF1R* antibody in xenograft tumor models. A predictive biomarker for IGF1R antibody response was not found.

Few studies have assessed the clinical efficacy of single agent anti-IGF1R antibody therapy in patients with OS [25]–[28]. Tumor response to anti-IGF1R therapy has only been published for a total of 7 patients with OS, and 4 of these patients had stable disease reported for at least 3 months after initiation of treatment [25], [26]. Additional phase I and phase II studies have been conducted but tumor responses in patients with OS have yet to be reported in these trials [27], [28]. Recent clinical trials have combined anti-IGF1R antibody with mammalian target of rapamycin (mTOR) inhibitors after *in vitro* and *in vivo* studies combining the two agents in sarcoma cells demonstrated decreased activation of pAKT [29], [30]. These clinical trials have included more than 50 patients with OS; however, while some patients had stable disease on treatment, the efficacy of combining these agents in OS is not clear [31], [32]. IGF1R expression in resected tumors was not found to be a predictive biomarker for OS response to combination therapy with an anti-IGF1R antibody and an MTOR inhibitor [31]. Analyzing these results are complicated by use of different IGF1R antibodies which may not be identical.

The efficacy and safety of anti-IGF1R antibodies have been explored in a wide variety of malignancies including bone and soft tissue sarcomas, squamous cell carcinoma, pancreatic cancer, lymphomas, colorectal cancer, and non-small cell lung cancer (NSCLC) [33]–[38]. While numerous studies have shown anti-IGF1R antibodies are fairly well-tolerated, few studies have shown the agents have significant efficacy. Recent phase II and phase III studies in squamous cell carcinoma, NSCLC (NCT00596830 and NCT00673049) and metastatic colorectal cancer failed to show improvements in patient outcomes [34], [35]. While studies have suggested that anti-IGF1R antibodies have activity in subsets of patients, the lack of large-scale efficacy has led to decreased enthusiasm for conducting new clinical trials with these agents. There is some concern that anti-IGFR1 antibodies will soon no longer be available for clinical use.

Currently, no predictive biological biomarkers have been discovered in OS. Percent tumor necrosis following neoadjuvant chemotherapy, as determined by the Huvos grading system, is a prognostic biomarker, however, alterations in therapy for patients with poor necrosis have never been shown to improve outcomes [39]. Recent studies have supported the investigation of IGF1R as a potential predictive biomarker in patients with solid tumors. *In vitro* and *in vivo* studies in rhabdomyosarcoma and breast cancer reported *IGF1R* mRNA and protein expression could be used to identify tumors with increased sensitivity to anti-IGF1R therapy. These studies concluded that higher expression of *IGF1R* is associated with increased responsiveness to IGF1R antibody-mediated therapy [14], [40]. An additional study assessing the predictive value of IGF1R surface expression in bone tumor xenograft models via antibody-labeled Immuno-SPECT imaging demonstrated that elevated surface expression IGF1R is associated with increased response to antibody-mediated therapy [41]. In the current study we did not find any relationship between *IGF1R* mRNA expression or surface expression of IGF1R and response to therapy. Similarly, Kurmasheva et al. also did not find a clear correlation between IGF1R protein expression and tumor response to the IGF1R antibody CP-751,871 [30]. Interestingly, a xenograft model that did not show significant surface expression of IGF1R had a high response to anti-*IGF1R* therapy. This suggests that high surface expression of IGF1R may not be a requirement for response to anti-IGF1R therapy and the antibody may work via additional, undiscovered mechanisms to inhibit cancer cell proliferation. Our findings support the clinical studies that demonstrated tumor responsiveness to IGF1R antibody in patients whose tumors did not express IGF1R [31].

While this study did not identify a predictive biomarker for anti-IGF1R therapy in OS, not all components of the pathway were assessed. This study focused on mutations in the *IGF1R* gene, as well as aberrations in mRNA and protein expression of IGF1R, however, the downstream IGF1R signaling pathways were not assessed. IGF1R has the capacity to activate both Ras-MAPK and PI3K-AKT signaling cascades; pathways linked to cell proliferation and cell survival [23], [42]. Cao et al. demonstrated that phosphorylated AKT predicted tumor response to an anti-IGF1R antibody better than IGF1R expression in mice bearing human rhabdomyosarcoma xenografts [14]. Mice treated with anti-IGF1R antibody, whose tumors showed suppressed IGF1R, had progression of disease if p-AKT was still activated.

The lack of a clear correlation between IGF1R mRNA expression and cell surface expression in OS cells with response to anti-IGF1R therapy suggests alternative IGF signaling pathways may continue to stimulate cell proliferation. Recently, Avnet et. al described the relevance of insulin receptor isoform A (IR-A) and hybrid-receptors (HR), which consist of one α and one β subunit IR heterodimer and one α and one β subunit IGF1R heterodimer in osteosarcoma samples [43]. IR-A and HR are expressed in almost all osteosarcoma samples and studies have shown HR binds IGFI and IGFII with high affinity, leading to downstream signaling [43], [44]. Inhibition of both IGF1R and IR led to significantly decreased OS cell proliferation compared with inhibition of IGF1R alone. These studies highlight the complex nature of IGF receptor-ligand interactions and provide a potential explanation why it has been difficult to identify a biomarker in anti-IGF1R therapy in OS.

Another limitation of this study is the lack of clarity as to which is the most appropriate laboratory model to investigate potential biomarkers in OS. This study demonstrated mRNA expression levels and gene copy numbers varied greatly between human OS samples, xenograft models, and their corresponding cell lines. Given the large heterogeneity in OS tumor samples, as well as the heterogeneity between corresponding cell lines and xenograft models, it remains to be determined which model is most representative of the disease. In addition, the ability of cancer cell lines and xenograft models to predict clinical efficacy of anti-cancer agents in human clinical trials is not clear [45]. The study would have been strengthened by performing the above assays on samples from recent clinical trials in which patient response to treatment is known. Unfortunately those samples were not available.

Preclinical studies demonstrate anti-IGF1R antibodies inhibit tumor cell growth in xenograft models of osteosarcoma, however, mutations in the *IGF1R* gene and changes in expression of *IGF1R* do not appear to be the driving force behind response to anti-IGF1R antibody therapy. OS is a heterogeneous and complex malignancy and studies assessing the role of potential predictive biomarkers need to consider these complexities. Further pre-clinical studies assessing targetable molecular pathways critical to the proliferation and survival of OS cells should be explored [46].

## Supporting Information

Table S1 **IGF1R Sequencing Primers.**
(DOCX)
