# Supplementary material for: Insulin-Like Growth Factor 1 Receptor and Response to Anti-IGF1R Antibody Therapy in Osteosarcoma
Source: PLoS One. 2014 Aug 29;9(8):e106249. doi: 10.1371/journal.pone.0106249 (PMC4149550; doi:10.1371/journal.pone.0106249)
Supplement: Table S1 — IGF1R Sequencing Primers. (DOCX) [file pone.0106249.s001.docx]

**Table S1. IGF1R Sequencing Primers.**

| **Exon** | **Primer Sequence** |
| --- | --- |
| IGF-1R (exon1) F306 | AAGGGGAATTTCATCCCAAA |
| IGF-1R (exon1) R472 | AGGAAAAGTTCCCGCAGTG |
| IGF-1R (exon2) F96 | ACAGCGTCTGGTCCAGTGTT |
| IGF-1R (exon2) R576 | GATGACCAGGGCGTAGTTGT |
| IGF-1R (exon2) F397 | CACATCCTGCTCATCTCCAA |
| IGF-1R (exon2) R929 | GAAGGGAGGTCAAGGAGGAG |
| IGF-1R (exon3) F1043 | CGGTCTCATCTCCGTCTCTC |
| IGF-1R (exon3) R1219 | CACACAGACACCGGCATAGT |
| IGF-1R (exon4) F282 | CACGCTCTTCCTTTGTAGGC |
| IGF-1R (exon4) R867 | CGTGCCAATGGCTAAAAGAC |
| IGF-1R (exon5) F63 | GTCAAGCCAGGGAAGTGTGT |
| IGF-1R (exon5) R538 | CATGACTCCACCCAAGACCT |
| IGF-1R (exon6) F259 | GCTGCCATTGTTAACAGCAA |
| IGF-1R (exon6) R677 | ACGGTGTTTTGGATGCTGTC |
| IGF-1R (exon7) F924 | ACGAGAAAGCCACTGAGGAA |
| IGF-1R (exon7) R1273 | CCCATTCAAGGATCGAAAGA |
| IGF-1R (exon8) F605 | ATTCTGTAATGCCCGACTGG |
| IGF-1R (exon8) R1084 | TGAGCTTGCGAAGAAGTGTG |
| IGF-1R (exon9) F635 | CTGTTGGCTTGCCAGAGTATC |
| IGF-1R (exon9) R934 | CACAGGAATGAACGGTCACA |
| IGF-1R (exon10) F380 | GGCTTTCATTCCCACTCTTG |
| IGF-1R (exon10) R787 | TCACTTCACCGAACCTGTCA |
| IGF-1R (exon11) F740 | GAGGAAGGACATCCCTGTGT |
| IGF-1R (exon11) R1227 | GTTGTGAGGAAGGTGGCAAT |
| IGF-1R (exon12) F586 | GGGAACCCAAATCCAACTTT |
| IGF-1R (exon12) R964 | AGCAGCCTAGCTCTTTGCAC |
| IGF-1R (exon13) F232 | CTCCTGCATTCATGGGAAAT |
| IGF-1R (exon13) R603 | TTCACACACAGTGCCGGTAT |
| IGF-1R (exon14) F133 | TTGTTTGTTTGCCGTGTTCT |
| IGF-1R (exon14) R460 | TCAAACCACTCAGCCACAGA |
| IGF-1R (exon15) F655 | TGCTGGTGCATACTTTTCCA |
| IGF-1R (exon15) R1119 | TCCTGAAAGCTCCCGATCTA |
| IGF-1R (exon16) F445 | CATCGCCTCCTGGTATTCTC |
| IGF-1R (exon16) R886 | TGCAGCCAAGAACATACTGG |
| IGF-1R (exon17) F817 | TTCCTGAAAAGCCAAAATGC |
| IGF-1R (exon17) R1200 | ACCGGTGGAAATGAAAACTG |
| IGF-1R (exon18) F884 | CTCGAAAGAAATTGGCATGG |
| IGF-1R (exon18) R1335 | ACCGTGCCCAGTATCTGAAC |
| IGF-1R (exon19) F561 | CGTGTCTGTGTCTTGCCTTG |
| IGF-1R (exon19) R977 | AGCCCACTGACAACAGGAAC |
| IGF-1R (exon20) F236 | GCTCGGGATGTAAGAAGTGC |
| IGF-1R (exon20) R732 | CACAGAGACATTTGGCCTGA |
| IGF-1R (exon21) F718 | CTTGTATGCGGGAAACCACT |
| IGF-1R (exon21) R1199 | AAGGATCAGCAGGTCGAAGA |
